# Treatment Results of Endoscopic Transnasal Orbital Decompression for Graves’ Orbitopathy—A Single-Center Retrospective Analysis in 28 Orbits of 16 Patients

**DOI:** 10.3390/jpm12101714

**Published:** 2022-10-14

**Authors:** Krzysztof B. Poślednik, Katarzyna Czerwaty, Nils Ludwig, Marta Molińska-Glura, Anna Jabłońska-Pawlak, Piotr Miśkiewicz, Ireneusz Kantor, Karolina Dżaman, Anna M. Cyran, Mirosław J. Szczepański

**Affiliations:** 1 Department of Otolaryngology, Centre of Postgraduate Medical Education, 01-813 Warsaw, Poland; 2 Department of Oral and Maxillofacial Surgery, University Hospital Regensburg, 93053 Regensburg, Germany; 3 Department of Economics and Forest Technology, Faculty of Forestry and Wood Technology, Poznan University of Life Sciences, 60-624 Poznan, Poland; 4 Department of Ophthalmology, Medical University of Warsaw, 02-091 Warsaw, Poland; 5 Department of Internal Medicine and Endocrinology, Medical University of Warsaw, 02-091 Warsaw, Poland; 6 Legorreta Cancer Center, Department of Pathology and Laboratory Medicine, Brown University, Providence, RI 02912, USA; 7 Department of Biochemistry, Medical University of Warsaw, 02-091 Warsaw, Poland

**Keywords:** endoscopic transnasal orbital decompression, Graves’ orbitopathy, dysthyroid optic neuropathy, compressive optic neuropathy

## Abstract

Graves’ orbitopathy (GO) is an extrathyroidal manifestation of Graves’ disease (GD), which can be associated with corneal ulcerations or optic neuropathy in severe forms. Transnasal endoscopic orbital decompression (TEOD) is a surgical procedure performed in order to decrease the intraorbital pressure by removing part of its bony borders in cases with excessive mass in orbit. The aim of this study was to present the results and evaluate the efficacy of TEOD for GO. The retrospective study included 28 orbits (16 patients) who underwent TEOD from 2017 to 2020. Outcome was evaluated based on visual acuity improvement, clinical activity score (CAS) decrease, proptosis, and intraocular pressure (IOP) reduction. A preoperative best-corrected visual acuity (BCVA) increased from 0.69 ± 0.385 (mean ± standard deviation) to 0.74 ± 0.332 (*p* = 0.17) postoperatively. CAS decreased in 15 orbits postoperatively. Proptosis decreased from 22.89 ± 1.873 mm to 21.25 ± 2.053 mm (*p* < 0.05). IOP decreased from a preoperative 16.11 ± 3.93 mmHg to 14.40 ± 3.27 mmHg (*p* < 0.05) postoperatively. In addition, postoperative relief of exposure keratitis was observed. The analysis of development of iatrogenic diplopia revealed increasing in degree of diplopia. TEOD shows rare complications, but significant improvements in BCVA, CAS, proptosis, and IOP.

## 1. Introduction

Graves’ orbitopathy (GO), also known as thyroid eye disease, is characterized by an autoimmune reaction involving the soft tissues of the orbit, leading to an increase in volume of orbital fat and muscles. It is the most common extrathyroidal manifestation of Graves’ disease (GD) as it affects 25–50% of patients. Rarely, the disease may also present in euthyroid patients or even hypothyroid as a result of Hashimoto thyroiditis. The active phase of the disease, associated with inflammatory infiltration and expansion of orbital muscles and adipose tissue is followed by an inactive phase, when the enlarged muscles and fat become fibrotic [1]. GO varies in severity with most cases being mild and self-limiting. More pronounced manifestations of GO include proptosis, eyelid retraction, diplopia, and exposure keratitis. In approximately 3–7% of patients with GO, compressive optic neuropathy (CON) develops, which poses a threat to eyesight [2]. In line with the Consensus Statement of the European Group on Graves’ Orbitopathy (EUGOGO), the first line of treatment is intravenous steroid therapy. Classical indications for surgical decompression include sight-threatening and disfiguring proptosis in patients with inactive GO [3]. Various surgical techniques for orbital decompression, involving the removal of orbital walls or—in selected patients—excessive fat tissue, have been developed. However, no single approach has unequivocally surpassed others. Our objective was to evaluate the effectiveness of transnasal endoscopic orbital decompression (TEOD) in GO.

## 2. Material and Methods

Approval of the local Ethics Committee was obtained for this study (#16/PB/2018 to I.K). We carried out a retrospective review of all cases of GO referred to our institution for transnasal endoscopic orbital decompression between 2017 and 2020. All surgeries were carried out by a single surgery team (M.J.S., K.B.P., K.C.). Prior to surgery, blood levels of anti-TSHR antibodies were tested, and the complete ophthalmologic status was determined. The ophthalmological evaluation included Hertel’s exophthalmometry, measurement of intraocular pressure (IOP), as well as evaluation of best-corrected visual acuity (BCVA), exposure keratitis, and diplopia. The ophthalmologic status was re-evaluated at 3-month follow-up. Clinical activity score (CAS), as described by the EUGOGO, was used to assess the activity of the disease. It includes the following clinical criteria: spontaneous retrobulbar pain, pain on attempted up- or downgaze, redness of the eyelids, redness of the conjunctiva, swelling of the eyelids, inflammation of the caruncle and/or plica, eyelid oedema, and conjunctival oedema. A CAS score equal to or greater than 3 indicates active GO. The GO severity assessment according to EUGOGO was used to evaluate the surgical indication for TEOD.

### 2.1. Surgical Technique

**Figure 1:**
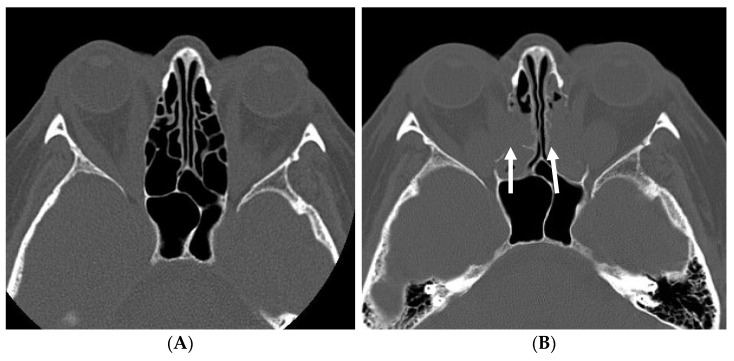
Axial CT scans showing orbital fat protruding into paranasal sinuses. Status before (**A**) and after (**B**) decompression. The fatty tissue filling the nasal cavity is visible (arrows).

An endoscopic transnasal procedure to achieve medial and inferior orbital wall decompression, leading to the herniation of orbital fat into the ethmoid and maxillary sinuses, was initially described by Kennedy et al. [4]. Here, the technique was modified to suit the individual indication and degree of proptosis. Following the induction of general anesthesia, an intravenous antibiotic was administered. The sinonasal mucosa was infiltrated with 1% lidocaine with adrenaline. Orbital decompression was immediately preceded with ipsilateral comprehensive endoscopic sinus surgery, including uncinectomy, maxillary antrostomy, anterior and posterior ethmoidectomy, frontal sinusotomy, and sphenoidotomy. The lamina papyracea and orbital floor were carefully elevated and removed with blunt dissection. Medial and inferior longitudinal incisions were made in the periorbita to allow the medial rectus at the most posterior extent and the orbital fat to herniate into the sinonasal cavity, effectively decompressing the contents of the orbit. To avoid any compression on the orbital tissue no nasal packing was used. Preoperative and post-operative CT scans are shown in Figure 1, demonstrating the anatomical extent of orbital decompression.

### 2.2. Statistical Analysis

The statistical analysis was performed by professional statistician (M.M-G.). For the observed variables, appropriate descriptive statistics such as the mean, standard deviation (SD) and minimum and maximum were evaluated. The analyzed parameters were compared using Student’s *t* tests. Compliance with the normal distribution was assessed by Kruskal–Wallis test. The *p*-value < 0.05 was considered as statistically significant.

## 3. Results

Sixteen patients (28 orbits) underwent TEOD for GO in the reviewed period. The cohort was composed of 12 women and 4 men, with a mean age of 62 years (range 35–74). The most common clinical signs of GO in our patient group were retrobulbar pain, eyelid swelling or redness of eyelids. All patients were treated with systemic steroids prior to surgery. Twelve patients (75%) received bilateral and four (25%) received unilateral TEOD. Table 1 presents the baseline characteristics of the patient cohort. 

**Figure 2:**
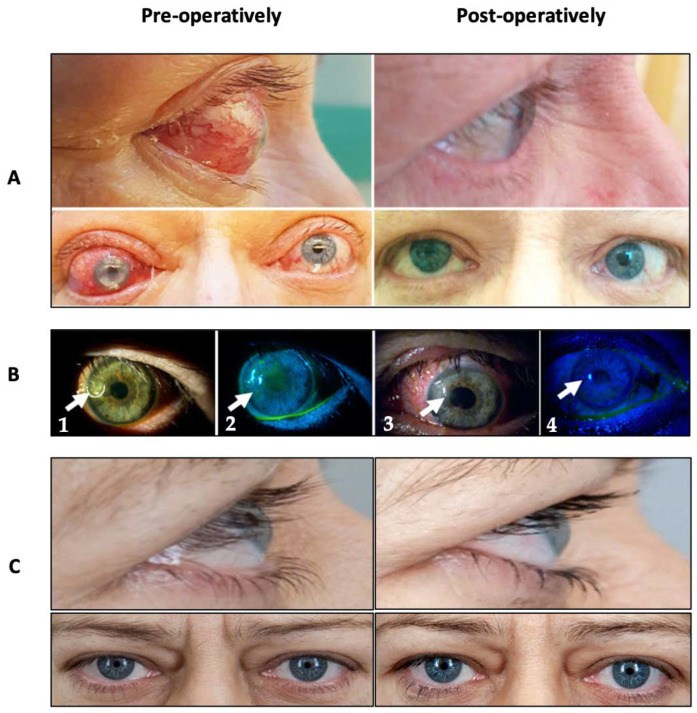
An ophthalmologic assessment of selected patients before and in the 3–12-month follow-up after TEOD. (**A**) The effect of treatment in a patient with exophthalmos and eyelid retraction. Patient still reports diplopia after 12 months after surgery. (**B**) The effect of treatment in a patient with corneal ulceration (arrows) before treatment (1,2) with ulceration and fluorescein binding at the site of ulceration, and after treatment (3,4) with no ulceration and no fluorescein binding at the site of the previous ulcer. Examination with diffuse illumination and 1% fluorescein with cobalt blue light at the slip lamp. (**C**) The effect of treatment in a patient with CON (right orbit).

At 3-month follow-up, significant improvement in BCVA from 0.69 ± 0.385 to 0.74 ± 0.332 was noted (*p* = 0.17). Similarly, proptosis decreased from a mean value of 22.89 ± 1.873 mm to 21.25 ± 2.053 mm (*p* < 0.05). Additionally, CAS decreased in 15 orbits (53.57%) postoperatively. IOP changed from 16.11 ± 3.93 mmHg in preoperative control to 14.40 ± 3.27 mmHg after surgery (*p* < 0.05). Moreover, marked amelioration of exposure keratitis was recorded in eleven cases (39.29%). However, we have seen statistically significant differences in the development of new-onset diplopia. No surgical complications were observed in our study. An overview of pre- and postoperative parameters is provided in Table 2. An ophthalmologic assessment of selected patients before and 3–12 months of the follow-up after TEOD is shown in Figure 2.

## 4. Discussion

GO profoundly impairs the quality of life of affected patients. Studies have consistently shown a deterioration in physical and mental health, as well as disturbances in social and work functions [5]. At the same time, only approximately 2% of patients with GO recover by both subjective and objective criteria [6]. 

The European task force on GO classifies the disease as mild, moderate-to-severe, and sight-threatening. Mild disease is usually treated with local measures unless the disease impact on the quality justifies immunosuppression or rehabilitative surgery. The operative measures may include decompression, squint and cosmetic eyelid surgery. Moderate-to-severe GO is characterized by lid retraction ≥2 mm, exophthalmos ≥3 mm, transient or permanent diplopia and corneal exposure. The first line of treatment in the active phase is intravenous glucocorticoid therapy. This view was confirmed in a randomized controlled trial on patients with active GO and CON, which showed that immediate surgery did not result in a better visual acuity [7]. In cases of insufficient response to glucocorticoids, orbital radiotherapy or immunosuppression with cyclosporine or rituximab may be indicated [3]. Urgent surgical decompression is reserved for cases of rapid or significant impairment of vision and corneal exposure, unresponsive to previous treatment. 

The number of surgical decompressions has increased over the past 20 years [8]. The propagation of endoscopic techniques resulted in broadening of the indication spectrum. In a systematic review of treatment outcomes of orbital decompression, the most common indication was a cosmetic reduction in proptosis (42.4%), followed by CON (40.6%) [9]. Importantly, patients in the non-inflammatory phase of the disease, in whom proptosis is the dominant clinical feature, may still experience headaches, pressure pain and orbital discomfort associated with prolonged visual concentration due to decreased venous outflow and orbital congestion [10]. 

In this study, we have reviewed our experience with TEOD in patients with GO. The patient demographics in this study are mostly consistent with that in existing literature [11,12]. Reduction in proptosis is the most commonly cited outcome measure. Endoscopic decompression is a very flexible technique and can be adjusted to individual cases. The average values of proptosis reduction reported in literature vary between 2.07 and 8.2 mm [12]. Endoscopic medial and inferior wall removal yields by 1.63–4.6 mm on average. When greater reduction is required, combined endoscopic and external three-wall removal can be adopted [13]. In the current study, the mean proptosis reduction was 2 mm. The ultimate therapeutic goal was not to achieve maximal decompression, but an optimal outcome; minimizing the risk of diplopia and other complications. TEOD performed primarily for cosmetic reasons is often more conservative than in cases of visual impairment [9]. Obviously more extended decompression is connected with more orbital fat protrusion to the nasal cavity and that in turn to a higher degree of proptosis regression. Orbit decompression extension in our patients was planned according to the patient’s state. In patients with minimal proptosis, medial wall decompressions were performed, whereas patients with severe proptosis with corneal ulceration and/or optic neuropathy also underwent inferior wall decompression. In some patients, the degree of proptosis may not correlate well with disease severity, as poor compliance of the orbital septum leads to a pressure build-up on the optical apex despite clinically mild exophthalmos. 

Most studies report an improvement in visual acuity following a decompressive surgery [1,2]; however, the heterogeneity of data presentation makes direct comparison difficult. The mean period between pre- and postoperative control of visual acuity should be considered, as well as the range of the decompression. Further improvement of vision parameters is anticipated during the few months after surgery [14]. In a review of 56 studies by Leong et al., the average improvement in visual acuity was 0.16 [9], and the results obtained by us is a BCVA improvement from 0.69 preoperatively to 0.74 postoperatively. However, the above-mentioned study included also techniques with external (not only TEOD) approach to orbit walls. Michael et al. have shown that in patients with CON small reduction in proptosis gives a significant improvement in visual acuity [15]. 

Pronounced orbital muscle oedema at the orbital apex can lead to optic nerve compression at the annulus of Zinn. It is heralded by a deterioration in visual acuity, quality of color vision or visual field loss, optic nerve oedema, and afferent pupillary defect [3]. In such cases, classical TEOD can be extended to the optic canal wall and incision of the optical sheath. In our department in the aforementioned period, one patient (one orbit) needed optic canal decompression to be performed. The patient previously had multiple orbital decompressions, including external approaches to inferior and lateral walls, but still the vision acuity deterioration was observed.

In our study TEOD was performed in an inactive phase of the disease in most of the patients. We observed, however, a decrease in the CAS after surgery, as other investigators [16]. All of the patients in our trial had received anti-inflammatory treatment prior to surgery.

One of the challenges in the optimization of treatment results in GO is minimizing the risk of new-onset and ideally elimination of existing diplopia without the necessity of subsequent squint surgery. In our series, ten patients had intermittent/permanent diplopia prior to surgery. Two patients developed diplopia after TEOD and were subjected to ophthalmic intervention for strabismus correction 6 months later. Preservation of the inferomedial orbital strut is an important step to prevent excessive displacement of the globe during decompression surgery. The inferomedial strut is a structure formed anteriorly by maxillary bone, ethmoid lamina and posteriorly junction of the palatine and ethmoid bones [13]. A comparative study on patients undergoing two- and three-wall decompression showed a lower rate of new-onset, and in 36% cases, resolution of an already existing diplopia in the group of patients with spared strut [17]. The results are consistent with our observation. Excellent results utilizing this technique were reported by Kingdom et al., in a retrospective case series of 77 patients (114 orbits), in which no new cases of diplopia occurred: 10.4% of patients improved and 3.8% worsened [18]. Leaving the strut limits access to the orbital floor, which may be contraindicated when substantial soft tissue prolapse is required, especially in cases with impairment of vision. An alternative approach to avoid inferomedial prolapse of the globe is to provide extra support to the medial rectus muscle by leaving a 10 mm section of periorbita along the medial orbital wall [19]. An interesting study by Yao et al. demonstrated a combination of partial strut preservation and orbital sling in patients undergoing an endoscopic medial and external lateral wall decompression. The authors concluded that the periorbital sling did not affect proptosis reduction when the anterior segment of the inferomedial strut was spared [20]. The traditional therapeutic concept for rehabilitative surgery in GO is to first decompress the orbit, then proceed with squint surgery and, in the last step, carry out esthetic eyelid corrections. The focus on improving patient’s quality of life and minimizing the number of surgeries led to the development of combined decompression with blepharoplasty [21].

GO is often associated with an elevation of IOP, which- if left untreated- leads to glaucomatous damage. Approximately one in five patients with GO presents with ocular hypertension [22,23]. Orbital decompression is known to reduce IOP by reducing retrobulbar and episcleral venous pressure, although there is no direct relationship between the degree of decompression and decrease in IOP [22]. In our study, despite the mean IOP being within the norm [24] both pre- and postoperatively, our results show a small, but statistically significant decrease at 3-month follow-up.

In our case series, the postoperative course of all patients was uneventful. A recent systematic review of literature on TEOD identifies epistaxis as the most common postoperative complication overall, occurring in 29 of 443 patients across 12 studies [12]. In the same review, three cases of cerebrospinal fluid leak were noted and eight cases of frontal or maxillary sinusitis [12]. The endonasal endoscopic technique is less traumatic to the infraorbital nerve than transantral and external approaches [25]. In selected patients, this risk may be further reduced by a sparing removal of the medial portion of the orbital wall. The most feared complication—partial or total loss of vision—is rarely reported in the literature, and to the best of our knowledge, there is no reporting of vision loss following orbital decompression via the transnasal endoscopic approach. Lastly, a case–control study on 10 patients undergoing primary and revision endoscopic decompression revealed similar complication rates in both groups. These results suggest that in rare cases of refractory orbitopathy, endoscopic decompression remains a safe strategy [26].

This study has some limitations. Firstly, it presents a single-institution experience. Secondly, the sample size did not allow further stratification of patients depending on time from onset of symptoms to surgery or severity of symptoms, which would enable us to make more detailed statements regarding safety and effectiveness of TEOD in respective patients group. The study would be further strengthened by employing pre- and postoperative quality-of-life assessments. 

## 5. Conclusions

The ultimate goal of surgical therapy for GO is the reduction in proptosis, restoration of visual acuity and prevention or minimization of diplopia. Our results show the effectiveness and safety of TEOD in this clinical context. However, an unmet need for randomized, controlled trials on the subject persists. 

## Figures and Tables

**Table 1:** Characteristics of 16 patients (28 orbits) at surgery.

	Total Number n = 16
Sex:	
Female (%)	12 (75)
Male (%)	4 (25)
Age (years):	
Mean (range)	62 (35–74)
Median	64.5
Smokers (%)	10 (62.5)
Mean fT4 (pmol/L)	18.55
Mean aTSHR (IU/L)	19.07
Mean aTPO (U/mL)	116.97
Mean aTG (U/mL)	489.11
Preoperative corticosteroids treatment systematically (%)	16 (100)
Surgical indication:	
Moderate to severe GO (%)	8 (28.57)
Sight-threatening GO (CON and/or corneal break) (%)	20 (71.43)
Unilateral (%)	4 (25)
Bilateral (%)	12 (75)

GO—Graves’ orbitopathy; CON—compressive optic neuropathy; CAS—clinical activity score; fT4—free thyroxine (normal range: 12.0–22.0 pmol/L); aTSHR-anti- thyrotropin receptor antibodies (normal range: <1 IU/L); aTPO—anti-thyroid peroxidase (normal range <  34 U/mL); aTG—thyroglobulin antibodies (normal range <  115 U/mL).

**Table 2:** Comparisons of preoperative and postoperative (3 months after TEOD) data of BCVA, proptosis and IOP.

	Pre-Op (Mean ± SD)	Post-Op (Mean ± SD)	*p* Value
BCVA	0.69 ± 0.38	0.74 ± 0.33	*=0.17*
Proptosis, mm	22.89 ± 1.87	21.25 ± 2.05	*<0.05*
IOP, mmHg	16.11 ± 3.93	14.40 ± 3.27	*<0.05*

BCVA = best corrected visual acuity; CAS = clinical activity score; IOP = Intraocular pressure; Pre-op = preoperative; Post-op = postoperative; SD = standard deviation.

## Data Availability

Not applicable.
